# Supplementary material for: Dietary Sodium and Potassium Intake: Data from the Mexican National Health and Nutrition Survey 2016
Source: Nutrients. 2022 Jan 11;14(2):281. doi: 10.3390/nu14020281 (PMC8779568; doi:10.3390/nu14020281)
Supplement: Supplementary file 1 [file nutrients-14-00281-s001.zip › Supplementary Table S1.pdf]

Supplementary Table S1. Description of the food groups used in the analysis of the food and beverage groups contributing to sodium and potassium intake

Food group	Food examples
1. Fruits	Fresh fruits, dried fruits and fresh fruit juices.
2. Vegetables	Fresh vegetables.
3. Eggs	Whole egg, white or yolk
4. Salt	Salt refers to the amount reported in the preparations.
5. Vegetable and animal fats	Margarine, butter, mayonnaise, lard and seeds oils.
6. Processed meats	Sausage, ham, dried meat and other industrialized meats.
7. Poultry	Any piece or organ of the chicken, turkey or pigeon.
8. Salty snacks	Popcorn, chips, japanese peanuts.
9. R-to-E Cereals*	Cereal box and cereal bars.
10. Carbonated SSBs*	Soft drinks.
11. Non-carbonated SSBs*	sports drinks, industrialized juices, industrialized tea, concentrated syrups, industrialized fruit nectar.
12. Seasonings	high sodium seasonings and sauces (Knorr Suiza, soy sauce, garlic salt, etc.)
13. Coffee or tea	coffee or tea without sugar or with sugar.
14. Nutritive or nonnutritive sweeteners	Brown sugar, refined sugar and Splenda.
15. Water	Fresh or bottled water.
16. Red meat	Pork or beef.
17. Oily seeds	Almonds, hazelnuts, walnuts, sunflower seeds, peanuts, etc.
18. Industrialized fruits, vegetables, and legumes	Fruits, legumes and vegetables, in juice, canned, pure, etc.
19. Legumes	Beans, lentils, soybeans, chickpeas, broad beans
20. Dairy	Whole milk, Liconsa, skim milk, low-fat, light and cheeses.
21. Dressings	Mayonnaise and dressings.
22. Yogurt and milk-based drinks	Yogurt, drinking yogurt and soy-based drinks.
23. Fish and seafood	Fresh fish, shrimp, oysters, crab, octopus.
24. Canned tuna and sardines	Canned sardines and tuna.
25. Cereal based sweets	Industrialized cookies, cakes and donuts.
26. Cereals	Rice, white bread, whole wheat bread, potatoes, pasta, corn, whole wheat, crackers, etc.
27. Sweet bakery bread	Sweet bread from bakery.
28. Corn tortilla	Corn tortilla.
29. Industrialized corn tortilla or tostadas	Industrialized corn tortilla or tostadas.

30. Non cereal based sweets	Dried fruits, crystallized or in syrup, candies.
31. Root vegetables	Sweet potato, yellow potato, white potato, yucca.
32. Instant soup	Industrialized pasta soup or condensed soup.
33. Alcohol	Beer, vodka, rum, etc.
34. Fast food	Burgers, hot dogs, french fries, pizza, sandwiches.
35. Industrialized baby food	Industrialized foods made from chicken, fruits, vegetables for babies
36. Others	Miscellaneous (seasonings, vinegar, cream substitute, vanilla extract, etc.)

R-to-E Cereals, Ready to eat cereals. SSBs, Sugar-Sweetened Beverages.
