# Supplementary material for: Dietary Sodium and Potassium Intake: Data from the Mexican National Health and Nutrition Survey 2016
Source: Nutrients. 2022 Jan 11;14(2):281. doi: 10.3390/nu14020281 (PMC8779568; doi:10.3390/nu14020281)
Supplement: Supplementary file 1 [file nutrients-14-00281-s001.zip › Supplementary Table S2.pdf]

Supplementary Table S2. Sodium and potassium consumption and percent contribution to diet by mealtimes and place of consumption in the Mexican population: ENSANUT 2016

	Pre-Schoolchildren		Schoolchildren		Adolescents		Adults	
**Sodium**	**mg/day**	**% Contribution**	**mg/day**	**% Contribution**	**mg/day**	**% Contribution**	**mg/day**	**% Contribution**
**Eating times**								
Breakfast	402.2 ± 41.9	27.0 ± 1.7	633.6 ± 48.2	26.1 ± 1.2	1096.5 ± 241.6	24.7 ± 1.9	604.8 ± 64.4	20.9 ± 1.5
Lunch	215.7 ± 37.2	11.9 ± 1.4	333.1 ± 33.4	3.3 ± 0.4	430.8 ± 67.9	12.3 ± 1.3	556.6 ± 76.2	16.2 ± 1.5
Midmorning	54.2 ± 10.1	3.6 ± 0.5	100.1 ± 19.1	3.3 ± 0.4	137.6 ± 40.3	3.2 ± 0.6	101.4 ± 34.2	2.3 ± 0.5
Meal	446.6 ± 30.4	30.5 ± 1.6	1103.4 ± 211.4	31.5 ± 1.1	1104.4 ± 88.0	34.2 ± 1.9	1114.6 ± 82.2	36.4 ± 1.6
Midafternoon	95.4 ± 14.7	6.9 ± 0.8	139.7 ± 19.0	5.1 ± 0.6	263.7 ± 69.6	5.5 ± 1.0	71.4 ± 13.7	2.8 ± 0.6
Dinner	298.1 ± 36.9	20.1 ± 1.5	533.8 ± 49.7	20.9 ± 1.0	710.2 ± 47.2	20.2 ± 1.2	683.6 ± 61.4	21.3 ± 1.2
**Potassium**	**mg/day**	**% Contribution**	**mg/day**	**% Contribution**	**mg/day**	**% Contribution**	**mg/day**	**% Contribution**
**Eating times**								
Breakfast	356.6 ± 19.5	24.1 ± 1.0	500.2 ± 36.4	23.5 ± 0.9	730.8 ± 75.0	23.2 ± 1.2	765.7 ± 85.1	21.5 ± 1.0
Lunch	189.2 ± 31.5	10.2 ± 1.0	237.8 ± 20.5	11.2 ± 0.7	306.8 ± 40.2	10.7 ± 1.1	452.5 ± 58.7	13.8 ± 1.2
Midmorning	153.2 ± 17.8	8.5 ± 0.8	143.2 ± 16.3	5.9 ± 0.6	182.5 ± 50.1	4.5 ± 0.7	128.8 ± 23.0	3.6 ± 0.6
Meal	411.3 ± 33.3	26.4 ± 1.4	706.9 ± 72.1	30.3 ± 1.3	922.0 ± 57.8	34.0 ± 1.6	1208.8 ± 146.7	36.1 ± 1.3
Midafternoon	193.6 ± 19.0	11.4 ± 1.1	175.6 ± 17.5	7.5 ± 0.6	200.0 ± 28.2	6.4 ± 0.9	131.3 ± 15.6	4.4 ± 0.5
Dinner	311.7 ± 24.7	19.5 ± 1.1	492.0 ± 49.2	21.5 ± 1.1	624.6 ± 42.9	21.2 ± 0.8	713.4 ± 109.8	20.6 ± 1.3

Data represents means and standard errors
