# Supplementary material for: Dietary Sodium and Potassium Intake: Data from the Mexican National Health and Nutrition Survey 2016
Source: Nutrients. 2022 Jan 11;14(2):281. doi: 10.3390/nu14020281 (PMC8779568; doi:10.3390/nu14020281)
Supplement: Supplementary file 1 [file nutrients-14-00281-s001.zip › Supplementary Table S3.pdf]

Supplementary Table S3. Mean intake and percentage contribution of sodium and potassium according to the Nova classification in the Mexican population: ENSANUT 2016

	**Pre-Schoolchildren**				**Schoolchildren**					**Adolescents**					**Adults**				
	**mg/day**		**Contribution (%)**		**mg/day**		**Contribution (%)**			**mg/day**		**Contribution (%)**			**mg/day**		**Contribution (%)**		
**Sodium**																			
Unprocessed or minimally processed foods	249.9	14.3	19.9	1.1	385.4	21.4	19.0	0.8		507.3	31.1	17.0	0.9	1	552.6	30.9	20.9	1.2	3
Processed culinary ingredients	465.4	44.4	30.7	1.6	1095.8	217.4	31.0	1.3		1301.1	129.9	35.6	2.1		1272.6	105.4	40.1	1.5	1, 2
Processed foods	195.2	28.8	11.0	1.5	394.0	41.0	13.0	1.1		576.0	118.3	12.7	1.3		393.8	38.6	12.1	1.0	
Ultra-processed foods	601.6	48.6	38.4	1.9	968.7	68.6	36.9	1.6		1358.8	136.8	34.6	1.7		913.4	99.7	27.0	1.8	1, 2, 3
**Potassium**																			
Unprocessed or minimally processed foods	1091.1	59.4	67.6	1.8	1718.7	107.7	76.0	0.9	1	2247.8	148.6	75.3	1.3	1	2908.7	252.0	83.4	0.9	1, 2, 3
Processed culinary ingredients	24.4	4.0	1.6	0.2	29.9	4.1	1.4	0.2		55.3	9.7	1.9	0.2		56.2	7.7	2.2	0.4	
Processed foods	35.0	5.0	3.0	0.6	62.0	5.2	3.1	0.3		133.8	27.9	4.0	0.4		99.5	9.0	3.8	0.3	
Ultra-processed foods	465.1	42.7	27.8	1.8	445.1	39.3	19.5	0.9	1	529.7	45.9	18.7	1.0	1	336.1	47.4	10.7	0.7	1, 2, 3

Data represents means and standard errors
1 statistically significant difference in contribution vs Pre-Schoolchildren (p <0.05)
2 statistically significant difference in contribution vs Schoolchildren (p <0.05)
3 statistically significant difference in contribution vs Adolescents (p <0.05)
